# *IL24* Expression in Synovial Myofibroblasts: Implications for Female Osteoarthritis Pain through Propensity Score Matching Analysis

**DOI:** 10.3390/medicina60050741

**Published:** 2024-04-29

**Authors:** Naoya Shibata, Yoshihisa Ohashi, Ayumi Tsukada, Dai Iwase, Jun Aikawa, Manabu Mukai, Yukie Metoki, Yui Uekusa, Masashi Sato, Gen Inoue, Masashi Takaso, Kentaro Uchida

**Affiliations:** 1 Department of Orthopaedic Surgery, Kitasato University School of Medicine, 1-15-1 Minami-ku, Kitasato, Sagamihara 252-0374, Kanagawa, Japan; shibachoku999@gmail.com (N.S.); 44134413oo@gmail.com (Y.O.); amidesutarere9010@yahoo.co.jp (A.T.); daiiwase19760601@yahoo.co.jp (D.I.); jun43814@gmail.com (J.A.); m.manabu0829@hotmail.co.jp (M.M.); yukiemetoki0826@gmail.com (Y.M.); mtakaso@kitasato-u.ac.jp (M.T.); 2 Department of Immunology, Kitasato University School of Medicine, 1-15-1 Minami-ku, Kitasato, Sagamihara 252-0374, Kanagawa, Japan; 3 Research Institute, Shonan University of Medical Sciences, Nishikubo 500, Chigasaki 253-0083, Kanagawa, Japan

**Keywords:** interleukin-24, osteoarthritis, pain, myofibroblast, sex difference

## Abstract

(1) *Introduction*: Despite documented clinical and pain discrepancies between male and female osteoarthritis (OA) patients, the underlying mechanisms remain unclear. Synovial myofibroblasts, implicated in synovial fibrosis and OA-related pain, offer a potential explanation for these sex differences. Additionally, interleukin-24 (IL24), known for its role in autoimmune disorders and potential myofibroblast production, adds complexity to understanding sex-specific variations in OA. We investigate its role in OA and its contribution to observed sex differences. (2) *Methods*: To assess gender-specific variations, we analyzed myofibroblast marker expression and *IL24* levels in synovial tissue samples from propensity-matched male and female OA patients (each *n* = 34). Gene expression was quantified using quantitative polymerase chain reaction (qPCR). The association between *IL24* expression levels and pain severity, measured by a visual analog scale (VAS), was examined to understand the link between *IL24* and OA pain. Synovial fibroblast subsets, including CD45-CD31-CD39- (fibroblast) and CD45-CD31-CD39+ (myofibroblast), were magnetically isolated from female patients (*n* = 5), and *IL24* expression was compared between these subsets. (3) *Results*: Females exhibited significantly higher expression of myofibroblast markers (MYH11, ET1, ENTPD2) and *IL24* compared to males. *IL24* expression positively correlated with pain severity in females, while no correlation was observed in males. Further exploration revealed that the myofibroblast fraction highly expressed *IL24* compared to the fibroblast fraction in both male and female samples. There was no difference in the myofibroblast fraction between males and females. (4) *Conclusions*: Our study highlights the gender-specific role of myofibroblasts and IL24 in OA pathogenesis. Elevated IL24 levels in females, correlating with pain severity, suggest its involvement in OA pain experiences. The potential therapeutic implications of IL24, demonstrated in autoimmune disorders, open avenues for targeted interventions. Notwithstanding the limitations of the study, our findings contribute to understanding OA’s multifaceted nature and advocate for future research exploring mechanistic underpinnings and clinical applications of IL24 in synovial myofibroblasts. Additionally, future research directions should focus on elucidating the precise mechanisms by which IL24 contributes to OA pathology and exploring its potential as a therapeutic target for personalized medicine approaches.

## 1. Introduction

Osteoarthritis (OA) stands as a prevalent and debilitating joint disorder characterized by the progressive deterioration of articular cartilage, alterations in joint structure, and the emergence of a chronic inflammatory milieu within the synovium [1,2]. While OA affects both men and women, there exists a notable disparity in its prevalence and severity between the sexes [3,4]. Women exhibit a higher prevalence of OA compared to men, constituting approximately 60% of OA cases worldwide [5,6]. This gender disparity is particularly notable in the knee and hand joints [5]. For instance, in Spain, women face 3–5 times the risk of incident hand OA and 2–3.5 times the risk of incident knee OA [7]. Similarly, in Saudi Arabia and China, odds ratios for prevalent knee OA are 2.14 [8] and 1.76 [9], respectively, when comparing women to men. These findings are reinforced by a systematic review and meta-analysis indicating that the prevalence of knee OA is higher in women than in men, with the disparity increasing with each decade starting at age 40 [10].

The clinical manifestations of knee OA can vary widely, with pain in the knee joint being the most common symptom [11,12]. These impairments, often associated with pain, can significantly impact daily activities and quality of life. Treatment options range from lifestyle adjustments and exercise to pharmacological interventions like analgesics and anti-inflammatory drugs [11,12]. Studies indicate differences in the clinical manifestations and pain experiences between male and female OA patients [5,6,7,8,9]. Women tend to use both over-the-counter and prescription pain medications more frequently than men, possibly due to higher pain levels and functional limitations [13,14]. Even after accounting for various demographic and health-related factors, women with OA are more likely to be prescribed and use larger quantities of non-steroidal anti-inflammatory drugs (NSAIDs) than men [15]. Therefore, emerging insights into gender-specific pain mechanisms may help elucidate why pain treatments vary in effectiveness among women. However, the differences in pain mechanisms between men and women are not yet fully understood.

OA is a comprehensive joint disorder affecting various structures such as muscles, subchondral bone, cartilage, ligaments, and synovial tissue [16,17]. In a healthy joint, the synovium comprises a cellular intimal layer and a subintimal connective tissue layer [18,19]. The intimal layer contains two distinct cell types: type A macrophages and type B synovial fibroblasts, with macrophages constituting a smaller proportion of cells in the intimal layer of normal synovium. Synovial fibroblasts play a crucial role in producing hyaluronic acid, a glycosaminoglycan, and proteoglycan-4 (PRG4), a mucinous glycoprotein, which are essential constituents of synovial fluid vital for joint lubrication [20,21]. Nevertheless, synovial fibrosis contributes to the joint pain and stiffness observed in advanced OA [22,23]. Amidst the intricate milieu of OA pathology, synovial myofibroblasts, specialized fibroblasts acknowledged for their pivotal role in extracellular matrix (ECM) synthesis, have taken center stage as active participants in the synovial fibrotic processes [24,25,26]. Notably, the proportion of synovial myofibroblasts correlates with pain levels, highlighting their potential role in modulating the symptomatic aspects of OA [26]. While research has begun to shed light on the complex interplay of factors contributing to organ fibrosis, the role of sex hormones, particularly in premenopausal women, emerges as a crucial and intriguing facet. Existing literature suggests a protective effect in premenopausal women, evident in their reduced susceptibility to severe forms of fibrosis across multiple organs [27]. We speculate that myofibroblasts could explain sex differences in OA.

Interleukin-24 (IL24), a member of the IL-20 family of cytokines, has been implicated in various autoimmune disorders, including psoriasis, arthritis, and inflammatory bowel diseases [28,29,30]. Previous studies have identified elevated levels of IL24 in the serum of patients with OA, suggesting its potential involvement in degenerative joint conditions [29]. IL24 is produced by immune cells such as B cells, monocytes/macrophages, mast cells, and NK cells [31,32,33,34]. In addition to immune cells, IL24 expression has been observed in myofibroblasts [35,36] and plays a role in the fibrotic process [37]. Moreover, IL24’s connection to sex-specific differences in immune diseases [38] underscores the need to scrutinize its involvement in the sexual dimorphism observed in OA. The exploration of IL24 and myofibroblasts has the potential to unveil key insights into the nuanced differences in OA presentation between men and women.

Here, we investigated the differences in myofibroblast marker expression and IL24 levels between male and female OA patients, exploring their potential associations with OA pathology. By elucidating the molecular mechanisms underlying sex disparities in OA, we aim to contribute to the development of personalized therapeutic approaches tailored to the specific needs of male and female patients.

## 2. Materials and Methods

### 2.1. Patients

**Figure 1:**
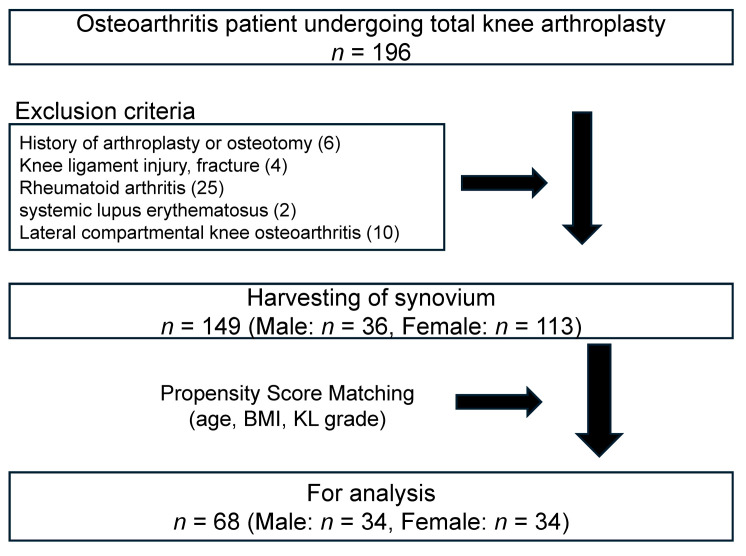
Flow of participant inclusion. Participants enrolled in or excluded from the current study. The values in parentheses indicate the number of excluded participants. BMI: body mass index; KL grade: Kellgren–Lawrence grade.

The research followed the guidelines set forth in the Declaration of Helsinki and received authorization from the Institutional Review Board at Kitasato University (protocol code: B19-259; approval date: 27 January 2020). All participants provided written informed consent, clearly indicating their willingness to take part in the study and permit the use of their synovial tissue post-surgery. The study cohort comprised male and female patients diagnosed with knee OA based on established clinical and radiographic criteria determined by the American Rheumatism Association [39]. A total of 196 OA patients underwent total knee arthroplasty (TKA) during the study period. We excluded 6 participants who had undergone arthroplasty or osteotomy, 4 participants with a history of knee ligament injury or fracture, 25 participants diagnosed with rheumatoid arthritis based on 2010 American College of Rheumatology/European League Against Rheumatism (ACR/EULAR) criteria [40], 2 participants diagnosed with systemic lupus erythematosus based on 2019 EULAR/ACR Classification Criteria [41], and 10 participants diagnosed with lateral compartmental knee OA. During TKA, synovial samples were obtained from 149 patients. After propensity score matching analysis, synovial samples from 68 participants were analyzed by PCR. Each of the 149 synovium specimens from female (*n* = 113) and male (*n* = 36) knee OA patients was promptly frozen in liquid nitrogen at −196 °C and then stored at −80 °C before RNA extraction. To create a matched cohort of male and female groups, we calculated a propensity score for each individual based on demographic characteristics (age, body mass index, and Kellgren/Lawrence grades) (Figure 1). The clinical characteristics of the patients are outlined in Table 1 according to their respective groups. Fresh samples from the female groups (*n* = 5) were employed to assess the gene expression in myofibroblasts.

### 2.2. Magnetic Isolation of Myofibroblast

To delineate the expression of IL24 in myofibroblast females, we utilized magnetic bead methods to isolate both the myofibroblast-rich fraction (MyoFb-RF) and fibroblast-rich fraction (MyoFb-PF). Fresh synovial membrane samples were immediately immersed in a collagenase solution with a concentration of 400 U/mL for a duration of 2 h at 37 °C to facilitate collagenase digestion. Cells were incubated for 30 min at 4 °C with biotin-conjugated anti-CD45 (pan-leukocyte marker) (Biolegend, San Diego, CA, USA), anti-CD31 (endothelial cell marker, eBiosciences, cat. no. 13-0319-82, San Diego, CA, USA). After two rinses with phosphate-buffered saline (PBS), the cells were treated with streptavidin-conjugated magnetic particles (BD Biosciences, San Jose, CA, USA) and introduced into a magnetic separation system (BD Biosciences) to segregate into a negative fraction (enriched with fibroblasts) and a positive fraction (containing fewer fibroblasts). Following this, the fibroblast-rich fraction underwent a 30 min reaction at 4 °C with PE-conjugated anti-CD39 antibody (BioLegend). Following two rinses with PBS, cells were subjected to anti-PE magnetic particles (BD Biosciences) for segregation into a CD39-positive (MyoFb-RF) and CD39-negative fraction (MyoFb-PF). MyoFb-RF and MyoFb-PF were then subjected to qPCR to examine IL24 and myofibroblast marker expression. Relative mRNA expression was determined according to the mean value calculated for the MyoFb-PF.

### 2.3. qPCR

The synovial tissue and cell samples were homogenized in TRIzol reagent (Invitrogen, Carlsbad, CA, USA) using a polytron homogenizer. Following homogenization, the samples were lysed in a mixture of 1 mL TRIzol and 0.2 mL chloroform, vortexed for 30 s, and then transferred to a MaXtract high-density tube (Qiagen, Valencia, CA, USA). After centrifugation (18,000× *g*, 4 °C, 5 min), the resulting aqueous phase was combined with an equal volume of isopropanol containing a precipitation carrier (Ethachinmate; Nippon Gene, Tokyo, Japan). The RNA pellet, obtained post-supernatant removal, underwent washing with 70% ethanol and centrifugation (18,000× *g*, 4 °C, 5 min). After discarding the supernatant, the RNA pellet was air-dried and dissolved in RNase-free water. Total RNA concentration was determined using a spectrophotometer (Denovix, Tokyo, Japan), with an OD 260/280 ratio greater than 1.8 considered suitable for qPCR analysis. Gel electrophoresis confirmed distinct bands of 28S and 18S. Subsequently, 1 μg of total RNA underwent cDNA synthesis using Superscript III, following the manufacturer’s protocol (Invitrogen). Primer sequences for *IL24* and myofibroblast-related markers (*MYH11*, *ET-1*, *ENTPD2*) [26,42,43] can be found in Table 2. The primer sequences were determined utilizing primer blast software (National Center for Biotechnology Information, Bethesda, MD, USA; https://www.ncbi.nlm.nih.gov/tools/primer-blast/) (accessed on 5 March 2021), and all primers employed were oligonucleotide purification cartridge-purified and procured from Hokkaido System Science (Sapporo, Japan). Gene expression (Gene/GAPDH) was determined using the delta-delta CT method, with relative expression calculated based on the average gene expression (Gene/GAPDH) level in the male or MyoFb-PF group being set to 1.

### 2.4. Statistical Analyses

The statistical analyses were carried out using SPSS 25.0 (IBM Corp., Armonk, NY, USA). To establish a matched cohort of male and female OA patients, propensity scores were computed based on baseline clinical variables, age, and the proportion with Kellgren and Lawrence grade 3–4. Categorical variables were analyzed using the chi-square test. The normality of all data was assessed using the Shapiro–Wilk test. The comparison of gene expression between males and females was conducted using the Mann–Whitney test. The correlation between IL24 levels and pain scores (VAS), as well as age, was determined using Spearman’s correlation coefficient. A significance level of *p* < 0.05 was applied to determine statistical significance.

## 3. Results

### 3.1. Expression of Myofibroblast Makers and IL24 in ST from Male and Female Knee OA Patients

**Figure 2:**
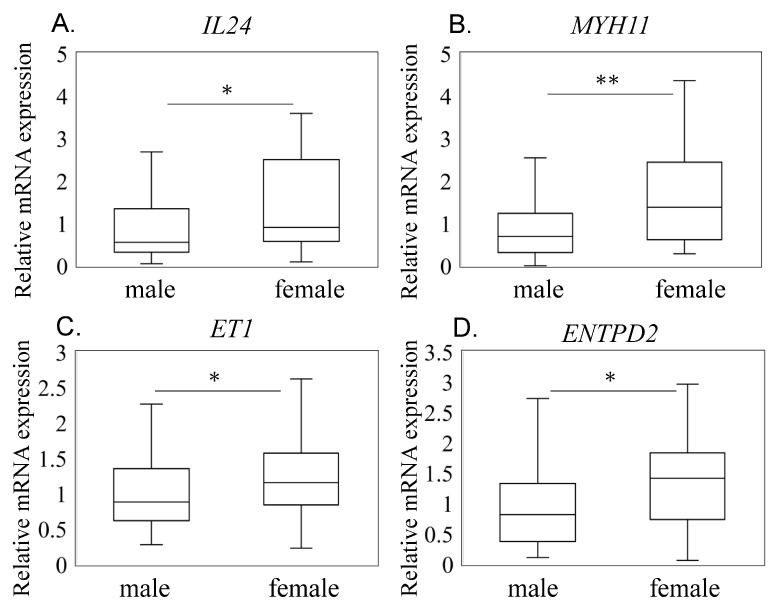
*IL24* and myofibroblast-related marker mRNA expression in male and female osteoarthritis patients. The expression levels of *IL24* (**A**), *ET-1* (**B**), *ENTPD2* (**C**), and *MYH11* (**D**) were assessed in synovial tissue samples obtained from male and female osteoarthritis (OA) patients. Box-and-whisker plots were used to depict the distribution of gene expression levels across male and female patients for each marker. The box represents the interquartile range (IQR), with the median indicated by the horizontal line inside the box. The whiskers extend to the minimum and maximum values within 1.5 times the IQR from the first and third quartiles, respectively. Statistical significance between male and female OA patients was determined using the Mann–Whitney test. * and ** indicate *p* < 0.05 and *p* < 0.01, respectively.

To ensure a balanced comparison, we utilized a propensity-matched cohort, matching for age, body mass index (BMI), Kellgren–Lawrence (KL) grade 3/4 ratio, and visual analog scale (VAS) score. The analysis of this matched cohort revealed no significant differences in these demographic and clinical characteristics between male and female patients (Table 1). Upon examining the expression levels of IL24, we observed a significant difference between male and female patients. Specifically, IL24 expression was significantly elevated in females compared to males (*p* = 0.011; Figure 2A), indicating a gender disparity in IL24 expression within the context of knee OA. Furthermore, we evaluated the expression of myofibroblast markers in synovial tissue samples from both genders. Interestingly, the expression levels of myofibroblast markers were notably higher in females compared to males. Specifically, the expression of ENTPD2 (*p* = 0.010), MYH11 (*p* = 0.002), and ET1 (*p* = 0.010) was significantly elevated in female knee OA patients relative to male patients (Figure 2C,D). These findings suggest that myofibroblast activity may be more pronounced in female knee OA patients compared to males.

### 3.2. Relationship between IL24 Expression and OA Pathology in Male and Female Knee OA Patients

**Figure 3:**
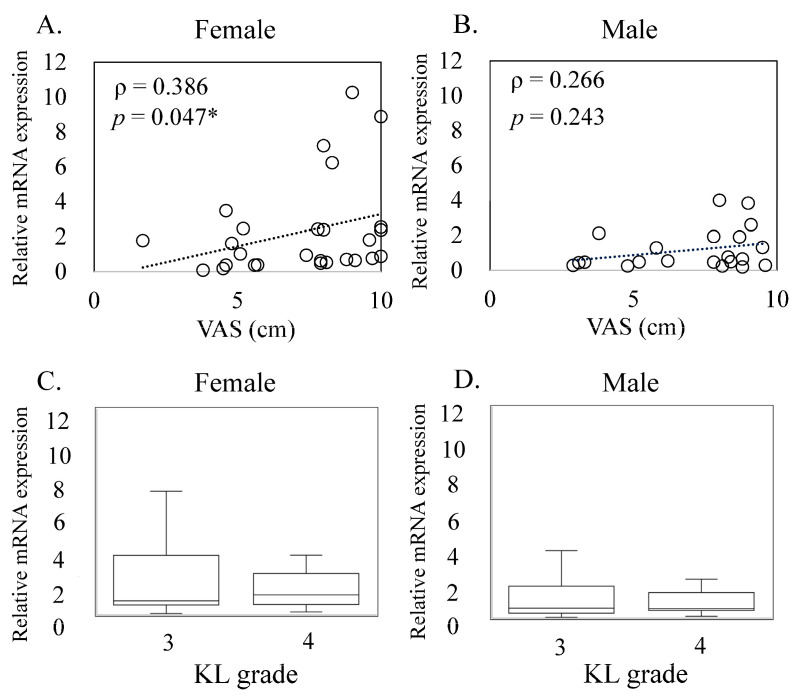
Relationship between IL24 mRNA expression and osteoarthritis pathology in male and female patients. This figure illustrates the correlation between *IL24* expression and pain levels as well as radiographic osteoarthritis (OA) grades (Kellgren–Lawrence grades, KL grades) in both male and female OA patients. Panels (**A**) and (**B**) depict the correlation between *IL24* expression and visual analog scale (VAS) scores in female and male patients, respectively. Statistical analysis was conducted using Spearman’s correlation coefficient, indicated by ρ. Statistical significance was defined as *p* < 0.05 (*). Additionally, *IL24* expression comparisons between KL3 and KL4 in female (**C**) and male (**D**) patients are provided.

Next, we examined the correlation between IL24 expression and pain severity. Interestingly, we found a significant positive correlation between IL24 expression and pain severity in females (ρ = 0.386, *p* = 0.047, Figure 3A,B). This suggests that higher levels of IL24 expression may be associated with increased pain severity in female knee OA patients. However, in male knee OA patients, we did not observe a significant correlation between IL24 expression and pain severity (ρ = 0.266, *p* = 0.243, Figure 3A,B), indicating that the relationship between IL24 expression and pain severity may differ between genders. Furthermore, we examined whether there was a difference in IL24 expression between patients with different degrees of OA severity, as classified by the Kellgren–Lawrence grading system. We found no notable distinction in IL24 expression between patients with KL3 and KL4 OA in both males and females (female, *p* = 0.959, male, *p* = 0.845, Figure 3C,D). This suggests that IL24 expression levels may not vary significantly with OA severity in either gender. A positive correlation was observed between *IL24* expression and pain severity in females (ρ = 0.386, *p* = 0.047, Figure 3A,B; however, no such correlation was identified in males (ρ = 0.266, *p* = 0.243, Figure 3A,B). Additionally, there was no notable distinction in *IL24* expression between patients with KL3 and KL4 in both females and males (female, *p* = 0.959, male, *p* = 0.845, Figure 3C,D).

### 3.3. Relationship between IL24 Expression and Age in Male and Female Knee OA Patients

**Figure 4:**
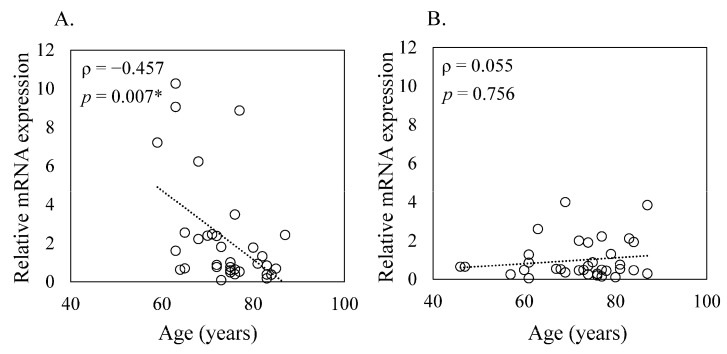
Relationship between *IL24* mRNA expression and age in male and female patients. This figure illustrates the correlation between *IL24* expression and age in both female (**A**) and male (**B**) OA patients. Statistical analysis was conducted using Spearman’s correlation coefficient, indicated by ρ. Statistical significance was defined as *p* < 0.05 (*).

The influence of hormonal changes associated with aging, particularly post-menopausal hormonal shifts in women, has been well-documented. Estrogen, in particular, has been shown to modulate the expression of IL24 [44]. Therefore, we next investigated the relationship between IL24 expression and age in male and female knee OA patients. We found a significant negative correlation between IL24 expression and age in females (ρ = −0.457, *p* = 0.007, Figure 4A). This suggests that as female knee OA patients age, there is a tendency for *IL24* expression to decrease. However, in male knee OA patients, we did not observe a significant correlation between *IL24* expression and age (ρ = 0.055, *p* = 0.756, Figure 4B). This indicates that age may not have a substantial impact on *IL24* expression levels in male OA patients.

### 3.4. Expression of IL24 in Myofibroblasts

**Figure 5:**
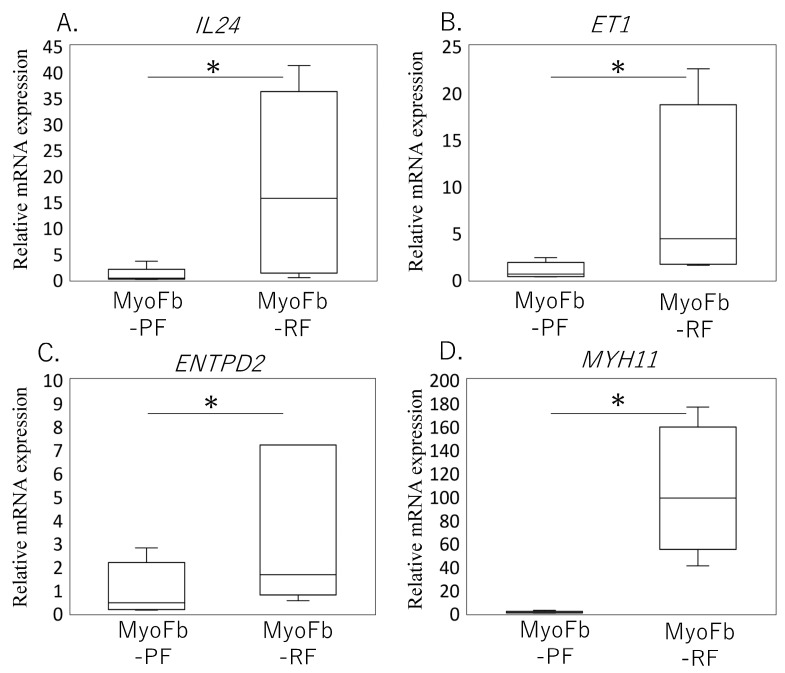
Expression of IL24 and myofibroblast-related markers in myofibroblast−rich and −poor fractions obtained from female osteoarthritic synovium. The expression levels of IL24 (**A**), ET1 (**B**), ENTPD2 (**C**), and MYH11 (**D**) were assessed in myofibroblast-rich and myofibroblast−poor fractions obtained from synovial tissue samples of female osteoarthritic (OA) patients. Box−and−whisker plots were utilized to illustrate the distribution of gene expression levels across the two fractions. The box represents the interquartile range (IQR), with the median indicated by the horizontal line inside the box. The whiskers extend to the minimum and maximum values within 1.5 times the IQR from the first and third quartiles, respectively. Statistical significance between myofibroblast−rich and myofibroblast−poor fractions was determined using the Wilcoxon test (* *p* < 0.05).

*IL24* expression in MyoFb-RF was significantly higher than in MyoFb-PF (*p* = 0.017, Figure 5A). Moreover, MyoFb-RF exhibited elevated expression levels of myofibroblast markers compared to MyoFb-PF. Specifically, MYH11 expression was significantly elevated in MyoFb-RF compared to MyoFb-PF (*p* = 0.028). Similarly, ET-1 expression was significantly higher in MyoFb-RF than in MyoFb-PF (*p* = 0.012). Additionally, ENTPD2 expression was notably higher in MyoFb-RF compared to MyoFb-PF (*p* = 0.046) (Figure 5B–D). These findings indicate that IL24 is predominantly expressed in myofibroblasts.

## 4. Discussion

The exploration of IL24 and myofibroblast markers in the context of our findings reveals a differential contribution for males and females. Here, we integrate our results with existing literature to elucidate the implications of IL24 in OA pathophysiology, emphasizing the gender-specific nuances and potential therapeutic avenues. Our study contributes to the growing body of evidence implicating myofibroblasts in the pathogenesis of female OA.

Previous research indicated that when synovial fibroblasts encounter increased stiffness in fibrotic environments, their functions shift away from their primary role of producing lubricating substances [24]. Lubricin, which is produced from the PRG4 gene, is widely recognized for its essential role in maintaining cartilage health [45,46]. Given that synovial fibroblasts are a major source of lubricin [47], a decrease in lubricin expression in fibrotic conditions might play a part in OA progression and pain. Synovial fibrosis, characterized by the excessive proliferation of synovial fibroblasts, their differentiation into myofibroblast-like cells, and the synthesis of ECM, may involve synovial myofibroblasts as contributors to fibrosis, playing a role in sustaining the chronic state observed in late-stage OA [25]. In the context of fibrogenesis, the synovium undergoes hypertrophy and forms fibrotic masses, contributing to the persistent joint pain and stiffness observed in OA [48,49]. A notable observation from our study is the gender-specific difference in IL24 expression, with higher levels detected in female OA patients compared to males. This aligns with previous studies highlighting the influence of IL24 on sex differences in immune diseases [38]. Notably, the increased expression of IL24 and myofibroblast markers, coupled with the identification of IL24 expression in synovial myofibroblasts, positions IL24 as a potential mediator of fibrotic conditions in female OA. The elevated expression in females may contribute to the heightened fibrotic responses observed in women, potentially offering insights into the gender disparities in OA pathology.

A notable aspect of our findings is the correlation observed between IL24 expression levels and pain scores, particularly in female OA patients. A recent study presented a murine model demonstrating sex differences in knee joint innervation and transcriptomic profiles of primary sensory neurons, with female mice having an earlier onset and greater hypersensitivity and pain behaviors [6]. A recent study has demonstrated spinal IL24’s contribution to neuropathic pain, with its inhibition through intrathecal IL24 neutralizing antibody injection shown to attenuate hyperalgesia [50]. Regulation of IL24 may be important for the development of tailored approaches to pain management in OA. The regulation of IL24 could indeed be crucial for developing tailored approaches to pain management in OA.

Previous studies have highlighted the complex relationship between pain and radiographic severity in knee OA [51,52,53]. In our study, IL-24 correlated with pain scores in women; however, it did not correlate with radiographic severity. While the radiographic severity of OA traditionally influences joint pain, there is growing evidence of discordance between pain and radiographic findings. The existence of a cause of pain that is independent of OA has been observed in women, while pain in men may be more dependent on joint space narrowing due to knee OA [52]. Another study reported that both radiographic severity and centrally-mediated symptoms were independently and significantly associated with pain severity in women with knee OA [53]. Central sensitization in knee OA is especially apparent among patients with reports of high levels of clinical pain despite the absence of moderate-to-severe radiographic evidence of pathologic changes in knee OA [51]. Importantly, IL-24 has been implicated in the process of central sensitization [50], with elevated IL-24 levels contributing to pain associated with central sensitization in radiographic OA.

The observed sex difference in OA, with women often experiencing more severe structural disease and symptoms compared to men, suggests a complex interplay of biological factors. Post-menopausal women, in particular, exhibit a greater disparity between the sexes, attributed partly to the decline in estrogen levels after menopause. Estrogen has been implicated not only in the pathogenesis of OA but also in the modulation of pain associated with the condition [54]. Interestingly, a study has shown that estrogen has the ability to significantly decrease the expression of IL24 in decidual stromal cells during early human pregnancy [44]. However, our study revealed an unexpected finding: IL24 expression in synovial tissue of female patients decreased with aging. The variation between genders, particularly more pronounced at post-menopausal stages, suggests that the decrease in estrogen levels following menopause is considered a potential contributor to the heightened risk of OA in women [55]. Despite extensive observational studies and clinical trials, conclusive evidence regarding this proposed mechanism remains elusive [56]. This suggests that the correlation between IL24 expression and pain in women may involve mechanisms independent of estrogen. To fully understand the relationship between IL24 and pain in OA, further investigation is warranted.

Drawing parallels with studies in RA and spondyloarthropathy, where IL24 is elevated in plasma and synovial cells, reinforces the notion of IL24 as a common factor in autoimmune joint disorders [28,29]. The potential of IL24 as a therapeutic target, demonstrated in a murine collagen-induced arthritis model through IL-20 receptor blockade [57], presents a promising avenue for intervention in OA. Insights from autoimmune joint disorders may inform targeted strategies to modulate IL24 levels and mitigate inflammatory responses in OA. Considering the gender-specific differences in IL24 expression and its correlation with pain severity, our findings have implications for gender-specific therapeutics in OA. Tailoring interventions based on the molecular profiles of male and female patients, with a focus on IL24 modulation, may pave the way for more effective and individualized treatment strategies. Precision medicine approaches that consider the specific contributions of IL24 could revolutionize the management of OA.

Recognizing the limitations of our study, such as the cross-sectional design and relatively small sample size, prompts consideration for future directions. Longitudinal studies with larger cohorts can provide insights into the dynamic changes in IL24 expression over time and its correlation with disease progression. Mechanistic studies exploring the downstream signaling pathways and functional consequences of IL24 in synovial myofibroblasts are essential for a more comprehensive understanding.

## 5. Conclusions

Our study highlights the multifaceted role of IL24 in OA pathophysiology, particularly in gender-specific differences and its correlation with pain severity. The elevated expression of IL24 in females compared to males underscores potential gender-specific mechanisms underlying OA progression. Additionally, the positive correlation between IL24 expression and pain severity in females suggests its involvement in pain modulation pathways in OA. However, the lack of correlation between IL24 expression and OA severity, as indicated by Kellgren–Lawrence grades, warrants further investigation into its specific roles in disease progression. These findings emphasize the importance of considering gender-specific factors in OA research and the potential of IL24 as a therapeutic target. Further mechanistic studies are needed to elucidate the exact role of IL24 in OA pathogenesis and its clinical implications for personalized medicine approaches.

## Figures and Tables

**Table 1:** Clinical characteristics in propensity-score-matched female and male osteoarthritis patient cohorts.

	Female (*n* = 34)	Male (*n* = 34)	*p*
Age (years)	73.9 ± 7.3	72.1 ± 10.3	0.410
BMI (kg/m^2^)	26.1 ± 4.4	26.3 ± 3.2	0.889
KL grade (3/4), N	14/20	12/22	0.402
VAS	7.2± 2.3	7.0 ± 2.3	0.700

BMI, body mass index; KL grade, Kellgren–Lawrence grade; VAS, visual analog scale.

**Table 2:** Primer sequences used for this study.

Primer	Sequence (5′–3′)	Product Size (bp)
*IL24*-F	GCCTCTGGATGCTGTGAAGA	200
*IL24*-R	ACTAAGCACCCATCCACTGC	
*ET1*-F	CTCTGCTGTTTGTGGCTTGC	103
*ET1*-R	GGACTGGGAGTGGGTTTCTC	
*ENTPD2*-F	CCCTCAAGTATGGCATCGTC	72
*ENTPD2*-R	CTGCCGGCCACTTGTAGATA	
*MYH11*-F	GATCGAGAACCTCACCCAGC	182
*MYH11*-R	TCTCCTCGGCTAACAACTGA	
*GAPDH-F*	TGTTGCCATCAATGACCCCTT	202
*GAPDH-R*	CTCCACGACGTACTCAGCG	

## Data Availability

The data presented in this study are available upon request from the corresponding author.
